# Predictors of tuberculosis treatment success among HIV-TB co-infected patients attending major tuberculosis treatment sites in Abeokuta, Ogun State, Nigeria

**DOI:** 10.11604/pamj.supp.2019.32.1.13272

**Published:** 2019-01-22

**Authors:** Kayode Akanbi, Ikeoluwapo Ajayi, Samuel Fayemiwo, Saheed Gidado, Abisola Oladimeji, Peter Nsubuga

**Affiliations:** 1 Nigeria Field Epidemiology and Laboratory Training Program, Haile Selassie, Asokoro, Abuja, Nigeria; 2 Department of Medical Microbiology and Parasitology, College of Medicine, University of Ibadan, Nigeria; 3 Department of Epidemiology and Medical Statistics, Faculty of Public Health, University of Ibadan, Nigeria; 4 Global Public Health Solution, Atlanta, Georgia, United States of America

**Keywords:** Tuberculosis, HIV, treatment outcome, Abeokuta, Nigeria

## Abstract

**Introduction:**

tuberculosis (TB) is the commonest opportunistic infection and cause of death in patients with Human Immunodeficiency Virus (HIV) in developing countries. World Health Organization (WHO) recommends 85% treatment success rate for all TB cases as an indicator of TB control. The study aimed at determining TB treatment success rate among TB-HIV co-infected patients and identifying predictors of successful treatment among patients in TB treatment sites in Abeokuta, Nigeria.

**Methods:**

it was a cross-sectional study among HIV-TB co-infected patients in the two major health facilities in Abeokuta, Nigeria. Socio-demographic characteristics with treatment history were obtained using a semi-structured questionnaire. Sputum samples were collected and tested for acid-fast bacilli (AFB) using a standard method according to national guideline for TB treatment to determine treatment success rate. Treatment success was defined as any HIV positive patient with a diagnosis of TB by acid-fast bacilli (AFB) smear positivity at diagnosis, who after 6 months of complete treatment becomes smear negative. Adjusted odds ratio was used to identify independent predictors of successful treatment outcome with confidence interval set at 95% and level of significance set at P < 0.05.

**Results:**

a total of 109 HIV-TB co-infected patients were enrolled for this study. Fifty-nine (54.1%) were females, 106 (97.3%) were newly treated for TB. Eighty-five (78.0%) were treated in a private health facility. A total of 91 had successful treatment outcome with a treatment success rate (TSR) of 83.5%. Eleven (10.1%) died, 5 (4.6%) defaulted, 1 (0.9%) failed treatment, 1 (0.9%) was transferred out. Successful treatment was associated with being newly registered (i.e. receiving TB treatment under the DOTS program for the first time), receiving TB treatment for the first time (adjusted OR = 18, 95%CI: 1.5-482.3) and being treated at a private health facility (adjusted OR = 14.1, 95%CI 4.27-48.4).

**Conclusion:**

treatment success rate of TB among HIV-TB co-infected patients in this study slightly falls below the WHO target. Registration status and health facility type were predictors of treatment outcome among study patients. Patients and healthcare workers in public facilities were educated on HIV-TB co-infection management.

## Introduction

Tuberculosis (TB) is an airborne infectious disease caused by *Mycobacterium tuberculosis* and primarily affects the lungs (pulmonary) but can also affect other parts of the body (extra-pulmonary) [1]. TB is curable if patients with drug-susceptible organisms are treated on time and are given sufficient uninterrupted therapy [2]. Despite the fact that TB is treatable and curable, it has proven difficult to eliminate, and this has been worsened by the HIV-AIDS pandemic [3]. TB is one of the most common infections that threaten people living with HIV (PLWH) in developing world because they are 26-31 times more likely to develop TB than persons without HIV [2]. There were 1.2 million HIV positive new TB cases globally in 2015 out of which 295,000 of the 400,000 global deaths occurred in Africa [3]. Nigeria, the most populous country in Africa with an estimated population of over 170 million people has a high HIV-positive TB death rate of 44 per 100,000 in 2014 [4]. It is believed that the TB situation in the country is HIV-driven [4]. TB deaths after treatment has been initiated remain high and occur within first few months of treatment [5]. A previous study showed that treatment success rates in hospitals in Nigeria varied widely between 43.7% and 86% [6]. The World Health Organisation (WHO) and the National Tuberculosis and Leprosy Control Programme (NTBLCP) currently recommend a case detection rate of 70% and a treatment success rate (TSR) of 85% for all TB cases [5]. It is believed that achieving these targets will lead to a reduction in TB prevalence, incidence, transmission and drug resistance to TB. Those who remain positive for active TB at 5 months after commencement of treatment (treatment failure) remain infectious for prolonged periods of time and this promotes further transmission of the disease in the community which ultimately leads to high rates of multidrug-resistant (MDR) TB especially in resource-limited settings [5, 6]. TB/HIV co-infected patients have multiple individual, disease specific and treatment related factors that can adversely affect their treatment outcomes. Some of these factors include: age, area of residence, treatment facility, type of treatment [2, 7]. Due to the significant diagnostic and therapeutic challenges that TB and HIV infection pose, this study was aimed to determine the predictors of TB treatment outcome and to assess the treatment success rate of TB among HIV patients attending major treatment sites in Abeokuta, Ogun State.

## Methods

**Study design:** we conducted a cross-sectional study between March 2014 and June 2015. Patients who were HIV positive were tested for pulmonary TB and those who tested positive for TB were recruited into the study and placed on anti-TB treatment for a minimum period of 6 months. The TB test was repeated 2 months after commencement of treatment and also 6 months after the commencement of treatment.

**Study setting:** we conducted this study in Abeokuta in the south-western part of Nigeria. Abeokuta is the capital of Ogun State with an estimated population of 451,607 [8]. We selected the Sacred Heart Hospital, Lantoro, Abeokuta, Ogun State, which is a privately owned Catholic Missionary General Hospital and the oldest existing medical hospital in Nigeria. The hospital has a 300-bed and acts as a referral point for many other hospitals in and around the State. The TB unit has 120 beds and it offers Directly Observed Treatment Short- course (DOTS) services and also serves as a reference center for the diagnosis and treatment of TB. We also selected State Hospital Ijaye, Abeokuta, Ogun State, which is a public hospital that belongs to the Ogun State Government and is under the State’s Hospital Management Board. The hospital has ≥ 200 staff and it provides DOTS services and is a reference center for the diagnosis and treatment of TB. The two selected hospitals usually register over 90,000 TB and non-TB related cases annually, provide DOTS services, have facilities to manage severe cases of TB infections and they manage over 80% of HIV/TB co-infected patients in the State. They are the two major treatment sites for TB in Ogun State and they are both recognized by the State National TB programme.

**Study population:** the study population were HIV patients with TB co-infection receiving care at the Sacred Heart Hospital, Lantoro, Abeokuta and the State Hospital, Ijaye, Abeokuta in Ogun State.

**Inclusion criteria:** patients who were HIV/TB co-infected and willing to receive treatment and were available for follow-up were recruited for the study.

**Exclusion criteria:** patients with multidrug MDR-TB and those who refused to give their consent directly or through their caregivers in the case of minors were excluded from the study.

**Sampling technique:** all the HIV/TB co-infected individuals who accessed the DOTS services of the two health facilities between March 2014 and June 2015 were recruited for the study.

**Data collection:** a semi-structured interviewer administered questionnaire was developed to capture socio-demographic information of the recruited patients. A structured data collection register was developed and used to record TB test results generated through the course of treatment.

**Laboratory methods:** two sputum samples from each of the consenting patients were collected into labeled and capped containers before transportation to the TB laboratory for processing. The sputa were tested for the presence of Acid Fast Bacilli (AFB) using the Ziehl Neelsen’s (ZN) staining technique. The sputa were also tested using Genexpert MTB-RIF automation method which detects *Mycobacterium tuberculosis* DNA and resistance to Rifampicin (RIF) by nucleic acid amplification technique (NAAT) [9].

**Data analysis:** we measured the following independent variables: age, sex, educational status, marital status, residence type, health facility type, registration status and treatment stage. The dependent variable measured in this study was the treatment outcome (successful and unsuccessful). Data entry, cleaning and analysis were done using Microsoft Excel (2008) and Epi-Info 7 (U.S. Centers for Disease Control and Prevention). Univariate data were summarized using frequencies, means and proportions. Multivariate analysis using logistic regression was done to determine the factors independently associated with treatment outcome. The level of statistical significance was set at p-value < 0.05.

**Ethical consideration:** ethical approval with reference number PH/200/31 was obtained from the ethics and research committee of the Ogun State Ministry of Health. Informed consent (written and verbal) was obtained from all respondents and in the case of minors, consent was obtained from caregivers. However, consent for participation of adolescent was granted by both the adolescent and their parents.

**Operational definitions:** treatment success was defined as the sum of patients who are declared ‘cured’ and those that have completed treatment. Previously treated patients were those who had undergone DOTS therapy but defaulted and came back for re-treatment. Newly registered patients are those who were receiving TB treatment under the DOTS program for the first time. A cured patient is one who was smear-positive at diagnosis, completed 6 or 8 months of treatment and who was smear-negative at the end of 6th or 7th month of treatment and at least one previous occasion. Multidrug-resistant TB (MDR-TB) is TB that does not respond to at least isoniazid and rifampicin, the two most potent anti-TB drugs.

## Results

**Figure 1:**
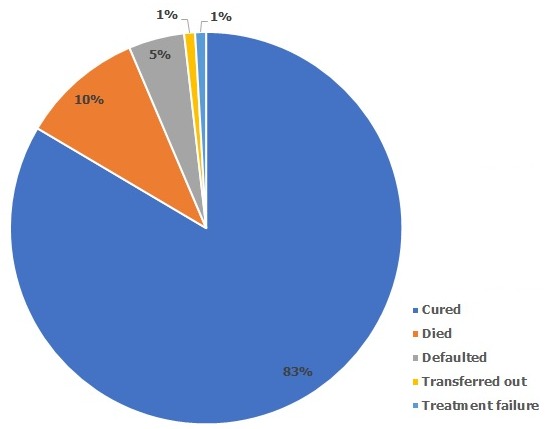
distribution of tuberculosis treatment outcome of HIV patients attending major treatment sites in Abeokuta, Ogun State - March 2014 to June 2015

A total of 109 HIV and TB co-infected individuals were recruited for the study. Fifty-eight (53.2%) were enrolled in Sacred Heart Hospital while 51 (46.8%) were enrolled in State Hospital Ijaye. Fifty (45.9%) of the respondents were males while 59 (54.1%) were females. The 30-39 age group has the highest number (36/109, 33%) of respondents. A total of 96 (88.1%) of the respondents lived in urban areas while 13 (11.9%) lived in rural areas. Forty-six (42.2%) had tertiary education and 73 (67.0%) were married (Table 1). Among the 109 patients recruited for the study, 91 had a successful treatment outcome with a treatment success rate (TSR) of 83.5%, 11 (10.1%) died, 5(4.6%) defaulted, 1 (0.9%) was transferred out and 1 (0.9%) had treatment failure (Figure 1).

**Table 1:** socio-demographic characteristics of HIV patients on tuberculosis treatment at major treatment sites in Abeokuta, Ogun State - March 2014 to June 2015 (N=109)

Variables	Frequency	Percentage (%)
Sex		
Male	50	45.9
Female	59	54.1
Age (years)		
< 10	11	10.0
10-19	4	3.7
20-29	21	19.3
30-39	36	33.0
40-49	26	23.9
50-59	8	7.3
≥ 60	3	2.8
Residence		
Urban	96	88.1
Rural	13	11.9
Educational Level		
No formal education	12	11.0
Primary	15	13.8
Secondary	36	33.0
Tertiary	46	42.2
Marital Status		
Married	73	67.0
Single	25	22.9
Divorced	11	10.1

In the bivariate analysis, patients who lived in urban areas were 3.9 times more likely to have a successful treatment outcome than those who lived in rural areas (p = 0.02). Also, patients who were treated in a private treatment facility were 14 times more likely to have a successful treatment outcome than those who were treated in the public treatment facility (p = 0.001). Patients who were newly registered and were accessing DOTS for the first time were more likely to have a successful treatment outcome than those who had been previously registered and were on DOTS, but they defaulted and returned for re-treatment. There was no evidence of an association between treatment outcome and sex (p = 0.09). Also, no evidence of an association existed between treatment outcome and level of education (p = 0.3) and age (p = 0.8) (Table 2). In the multivariable analysis, predictors of successful tuberculosis treatment outcome were among the HIV-TB co-infected patients recruited for the study were being newly registered for treatment (adjusted OR 18, 95%CI 1.5-482.2). Those who were newly registered and receiving TB treatment for the first time were 18 times more likely to have a successful treatment outcome. Those being treated in a private health facility are 14 times more likely to have a successful treatment (adjusted OR 14.1, 95%CI 4.3-48.4). However, residing in urban areas was found not to be a good predictor of treatment outcome i.e. not statistically significant (adjusted OR 3.9, 95%CI 0.9-16.5) (Table 3).

**Table 2:** variables associated with tuberculosis treatment outcome among HIV patients attending major TB treatment sites in Abeokuta, Ogun State - March 2014 to June 2015

Variables	Successful Outcome n (%)N= 91	Unsuccessful Outcome n (%)N=18	OR (95% C.I)	P value
**Sex**				
Male	45 (41.3)	5 (4.6)	2.5(0.8-7.7)	0.09
Female	46 (42.2)	13 (11.9)		
Residence				
Urban	83 (76.1)	13 (11.9)	3.9(1.1-14.1)	0.02
Rural	8 (7.3)	5 (4.6)		
**Registration status**				
Previously treated	1 (0.9)	3 (2.8)	0.05 (0.0-0.7)	0.01
New	90 (82.6)	15 (13.8)		
Facility Type				
Private	76 (69.8)	9 (8.3)	14.1 (4.8-4.1)	0.001
Public	9 (8.3)	15 (13.8)		
**Education**				
Not educated	24 (22.0)	3 (2.8)	1.7 (0.4-6.7)	0.3
Educated	67 (61.5)	15 (13.8)		
**Age (years)**				
< 25	14 (12.8)	3 (2.8)	0.9(0.2-3.5)	0.8
≥ 25	77 (70.6)	15 (13.8)		

OR = odds ratio; CI = confidence interval; p <0.05, Ref = the reference category Fisher’s exact P value used for cells <5

**Table 3:** predictors of successful tuberculosis treatment outcome of HIV patients attending major treatment sites in Abeokuta, Ogun State - March 2014 to June 2015

Variables	Successful Outcome n (%)N = 91	Unsuccessful Outcome n (%) N = 18	AOR (P value)	Confidence interval
**Sex**				
Female (ref)	46 (42.2)	13 (11.9)	1	
Male	45 (41.3)	5 (4.6)	0.33 (0.05)	0.11 - 0.009
Residence				
Rural (ref)	8 (7.3)	5 (4.6)	1	
Urban	83 (76.1)	13 (11.9)	3.9 (0.04)	0.9 - 16.5
**Registration Status**				
Previously (ref) treated	1 (0.9)	3 (2.8)	1	
New	90 (82.6)	15 (13.8)	18 (0.013)	1.5 - 482.3
**Facility Type**				
Public (ref)	9 (8.3)	15 (13.8)	1	
Private	76 (69.8)	9 (8.3)	14.1 (<0.001)	4.3 - 48.4
**Age (years)**				
< 25 (ref)	14 (12.8)	3 (2.8)	1	
≥ 25	77 (70.6)	15 (13.8)	1.1 (0.935)	0.3 - 4.1
**Education**				
Not educated	24 (22.0)	3 (2.8)	1	
Educated	67 (61.5)	15 (13.8)	2.7 (0.359)	0.3 - 22.0

## Discussion

This study was aimed at determining the predictors of TB treatment outcome and the treatment success rate of TB among HIV patients attending major treatment sites in Abeokuta, Ogun State. We found that receiving treatment in a private health facility was a predictor of having a successful TB treatment outcome. The successful treatment outcome may be because the private health facility in this study is a non-profit Catholic mission hospital offering free TB treatment services. Also, unlike the public health facility which also offers free TB treatment services, the private health facility is better structured and organized [10]. The healthcare workers in the private health facility exhibit a higher level of professionalism [10]. They do not embark on incessant industrial actions as experienced at the public health facility during the study which may cause the interruption of treatment and lead to unsuccessful treatment. The private health facility in this study had a more successful treatment outcome rate than the public health facility and it is consistent with the reports of Efegbere (2014) and Gidado (2009) [11, 12].

We also found that being newly registered for treatment (DOTS) and receiving the TB treatment for the first time is another predictor of having a successful treatment outcome. Newly registered patients are 18 times more likely to have a successful treatment outcome than those previously treated. Only one percent of those who were previously on treatment but defaulted and were back for treatment had a successful treatment outcome. A study carried out in South-eastern Nigeria by Ifebunandu in 2013 also showed that only 4% of the previously treated patients had a successful treatment outcome [6].

The TB treatment success rate among HIV-infected patients in this study was 83.5%. The TSR is high but slightly lower than the WHO and NTBLCP recommended TSR of 85%. However, it is higher than the TSR recorded before the introduction of DOTS program which varied widely between 43.7% and 71% [6]. The tuberculosis treatment success rate of 83.5% found in our study is slightly lower than the national target of 85. However, it is still higher than the 81% in Kano state, 71% in Cross River state, 80% in Ebonyi State, national value of 78% and 80% reported in Southern Ethiopia [1, 13]. The higher treatment success rate shown in Abeokuta,Ogun state in this study may, therefore, suggest good performance by the State’s TB program through the DOTS program.

### Study limitations

The culture of sputum samples to isolate *Mycobacterium tuberculosis* which is recommended by WHO and NTBLCP could not be carried out due to time constraint. However, we conducted Genexpert *Mycobacterium tuberculosis* (MTB) rifampicin (RIF) test on the patients’ sputum sample. This test gives a faster turn-around time and also detects rifampicin resistance MTB. The study was conducted within a mandatory period of 16 months to be able to meet up with academic timeline. Hence, there was no room a lengthy duration to be able to have an increased sample size.

## Conclusion

The tuberculosis treatment success rate among HIV-TB co-infected patients in Abeokuta, Ogun State is high but slightly below WHO target. Facility type and registration status (i.e being newly registered and treated or previously registered and treated) were predictors of successful tuberculosis treatment outcome among HIV-TB co-infected patients. The following public health actions were undertaken after the study, patients and healthcare workers in public health facilities in Ogun State were educated on HIV-TB co-infection management. Also, information, education and communication (IEC) materials were developed and shared among healthcare workers and HIV-TB co-infected patients in public health facilities across Ogun State. We recommended that defaulters among HIV-TB co-infected patients should be discouraged by individually (to ensure confidentiality and prevent stigmatization) counseling newly registered patients at the DOTS clinic on side effects and the risk associated with disrupting TB treatment.

### What is known about this topic

- HIV is one of the infections responsible for the increase of TB in Nigeria is HIV driven;
- TB is curable if patients with drug-susceptible organisms are treated on time and are given sufficient uninterrupted therapy;
- Achieving a treatment success rate of 85% will lead to a reduction in TB prevalence, incidence, transmission and drug resistance.

### What this study adds

- The treatment success rate of TB among HIV-TB co-infected individuals in Ogun State, Nigeria is 83.5%;
- HIV-TB co-infected individuals accessing TB treatment in private health facility in Abeokuta, Ogun State have increased chance of having a successful treatment.

## Competing interests

The authors declare no competing interests.
